# Appendiceal Neuroendocrine Tumors: Prognostic Role of Mesoappendiceal Invasion and Implications for Recommending Right Hemicolectomy versus Simple Appendectomy

**DOI:** 10.1245/s10434-025-17982-7

**Published:** 2025-08-05

**Authors:** Diamantis I. Tsilimigras, Pamela Lu, Susan Tsai, Timothy M. Pawlik, Bhavana Konda, Dipen Patel, Vineeth Sukrithan, Jordan M. Cloyd

**Affiliations:** 1 Department of Surgery, Division of Surgical Oncology, The Ohio State University Wexner Medical Center and James Comprehensive Cancer Center, Columbus, OH USA; 2 Department of Medicine, Division of Medical Oncology, The Ohio State University Wexner Medical Center and James Comprehensive Cancer Center, Columbus, OH USA

**Keywords:** Mesoappendiceal, Invasion, aNET, Neuroendocrine, Colectomy, Appendectomy

## Abstract

**Background:**

Current guidelines are conflicting as to whether mesoappendiceal invasion (MAI) among patients with appendiceal neuroendocrine tumors (aNETs) warrants right hemicolectomy (RHC), especially in the absence of other concomitant high-risk features.

**Methods:**

Patients who underwent resection of aNETs were identified in the National Cancer Database. Patients with pT3 aNETs (i.e. size > 4 cm or MAI/subserosal invasion [SI]+) were further stratified as pT3a (size ≤ 4 cm, + MAI/SI) or pT3b (size > 4 cm, ± MAI/SI). The association of MAI/SI with nodal metastasis (pN+) relative to the presence/absence of other risk factors was examined. The prognostic impact of the extent of resection (i.e. RHC vs. appendectomy) among patients with MAI/SI was assessed.

**Results:**

Among 4819 patients who underwent resection for aNETs, 1662 had pT3 tumors, of which 1309 (78.7%) were pT3a and 353 (21.3%) were pT3b. The overall incidence of pN+ disease was 7.5%, and varied by American Joint Committee on Cancer (AJCC) pT stage (pT1: 0.9%; pT2: 9.2%; pT3: 8.5%; pT4: 29.8%; *p* < 0.001). pT3a stage was less frequently associated with pN+ disease compared with pT3b disease (6.8% vs. 14.7%; *p* = 0.02). In the absence of other established risk factors, the presence of MAI/SI alone was associated with a low probability of pN+ (3.4%). The 3-year overall survival among patients with pT3a aNETs was comparable following RHC versus simple appendectomy (92.7% vs. 95.2%; *p* = 0.43).

**Conclusions:**

Among patients with resected aNETs, MAI/SI alone in the absence of other established risk factors was associated with a low likelihood of nodal metastasis and equivalent long-term outcomes regardless of the extent of surgical resection. The presence of MAI/SI alone should not be an indication for RHC.

**Supplementary Information:**

The online version contains supplementary material available at 10.1245/s10434-025-17982-7.

Appendiceal neuroendocrine tumors (aNETs) are rare neoplasms of the gastrointestinal tract, accounting for approximately 60% of all appendiceal tumors.^1,2^ aNETs are often diagnosed incidentally, with an estimated prevalence of 0.3–0.9% among patients undergoing appendectomy.^3^ Despite their rarity, the reported incidence of aNETs has increased in recent years, largely due to the widespread use of cross-sectional imaging and increased referral to specialized neuroendocrine tumor (NET) centers.^4,5^ Although aNETs are generally considered indolent neoplasms, the prognosis of affected patients is largely dependent on tumor size, histologic features (i.e., Ki67, mitotic index), and the presence of lymph node (LN) or distant metastases.^2^

To date, several criteria exist across different society guidelines to guide surgical decision making for patients with aNETs.^6–8^ While simple appendectomy is considered curative for small tumors (< 1 cm), most guidelines recommend completion right hemicolectomy (RHC) for aNETs >2 cm in size or in the setting of incomplete resection (R1/R2).^6–8^ Nevertheless, there remains a ‘gray zone’ regarding the management of aNETs measuring 1–2 cm, for which RHC is often recommended when one or more high-risk features are present. These high-risk features include high tumor grade, mesoappendiceal invasion (MAI), Ki-67 >2%, lymphovascular invasion (LVI), or perineural invasion (PNI).^8,9^ Due to the rarity of aNETs, most published data to guide surgical management are derived from relatively small and heterogeneous populations, limiting the strength of current evidence and contributing to variability in clinical practice.^10^

Among the identified high-risk features, MAI has been considered a key factor associated with increased risk of LN metastasis among patients with aNETs.^9,11^ While the European Neuroendocrine Tumor Society (ENETS) and North American Neuroendocrine Tumor Society (NANETS) guidelines recommend RHC for patients with aNETs 1–2 cm in size when MAI is present,^7,9^ the National Comprehensive Cancer Network (NCCN) guidelines do not explicitly define whether MAI alone warrants an RHC, especially in the absence of other concomitant high-risk features.^6^ To date, there is a paucity of data to inform the risk of nodal disease associated with MAI and whether or not the presence of MAI alone warrants an oncologic resection rather than simple appendectomy for patients with aNETs. Therefore, the objective of the current study was to characterize the probability of LN metastasis among patients with resected aNETs in the setting of MAI alone or in combination with other high-risk features. In addition, we sought to define long-term outcomes of patients with MAI treated with RHC versus simple appendectomy using data from the National Cancer Database (NCDB).

## Methods

### Data Source and Patient Selection

The NCDB is a comprehensive clinical oncology database comprising over 34 million individual cancer patient records. The NCDB collects data from over 1500 Commission on Cancer (CoC)-accredited medical facilities across the United States. The data repository captures details on more than 70% of index cancer cases on a national scale. The current study included individuals aged 18 years and older who underwent resection of aNETs. Diagnosis of an aNET was based on the International Classification of Disease for Oncology, Third Edition (ICD-O-3) topographical code C18.1, and ICD-O-3 morphological codes 8240 through 8249. Surgical procedures were categorized as appendectomy/segmental resection (codes 30, 32), RHC (codes 40, 41), and total colectomy/other (codes 50, 60, 70, 80, 90), as previously described.^10,12^ Patients with unknown pathologic T stage (pTx), pT0 or pTis disease, and individuals with metastatic tumors were excluded from the current analysis. In addition, given that information on MAI (i.e., primary independent variable) was not available in American Joint Committee on Cancer (AJCC) staging manuals prior to the 8th edition, the data were limited to individuals undergoing resection in 2018 or thereafter. The study was determined to be exempt by the Institutional Review Board of the Ohio State University Wexner Medical Center.

### Variables and Outcomes of Interest

Variables of interest included patient age, sex, race, Charlson–Deyo score, facility type (i.e., community cancer program, comprehensive community cancer program, academic/research program, integrated network cancer program), pathologic AJCC 8th edition T stage (pT), pathologic N status (pN; i.e., negative or positive), tumor size, number of sampled nodes, number of positive nodes, LVI, tumor differentiation, type of surgery (i.e., appendectomy, RHC, total colectomy/other), and surgical margins (i.e., R0, R1/R2). The primary independent variable was the presence of MAI or subserosal invasion (SI). To delineate the role of MAI/SI, pT3 aNETs (defined as tumors >4 cm, or tumors with MAI/SI according to the AJCC 8th edition staging system) were substratified as pT3a (tumor size ≤ 4 cm and definitive presence of MAI/SI) or pT3b (tumor size > 4 cm, ± MAI/SI) [electronic supplementary material (ESM) Table 1]. Primary outcomes of interest were pathologic LN metastasis (pN+) and overall survival (OS). OS was defined as the time elapsed between aNET resection and death or last follow-up.

**Table 1:** Clinicopathologic characteristics of patients undergoing resection for aNETs

Variables	Total [*n* = 4819]
Age, years [median (IQR)]	51 (34–63)
Sex	
Female	2869 (59.5)
Male	1950 (40.5)
Race	
Non-Hispanic White	4164 (86.4)
Non-Hispanic Black	411 (8.5)
Hispanic/other	244 (5.1)
Charlson–Deyo Score	
0–1	4520 (93.8)
≥ 2	299 (6.2)
Facility type	
Community Cancer Program	291 (6.0)
Comprehensive Community Cancer Program	1343 (27.9)
Academic/Research Program	887 (18.4)
Integrated Network Cancer Program	727 (15.1)
Not available	1571 (32.6)
8th edition AJCC pT stage	
pT1	2318 (48.1)
pT2	239 (5.0)
pT3	1662 (34.5)
pT4	600 (12.4)
pN status	
pN0	2084 (43.3)
pN+	362 (7.5)
Unknown/not examined	2373 (49.2)
Tumor size, cm [median (IQR)]	0.9 (0.4–1.6)
Number of sampled nodes [median (IQR)]	0 (0–16)
Number of positive nodes [median (IQR)]	0 (0–0)
Lymphovascular invasion	568 (11.8)
Tumor differentiation	
Well	3418 (70.9)
Moderate	387 (8.0)
Poor	282 (5.9)
Unknown	732 (15.2)
Type of surgery	
Appendectomy/segmental resection	2910 (60.4)
Right hemicolectomy	1498 (31.1)
Total colectomy/other	411 (8.5)
Surgical margins	
R0	4555 (94.5)
R1/R2	176 (3.7)
Unknown	88 (1.8)

Data are expressed as *n* (%) unless otherwise specified

*aNETs* appendiceal neuroendocrine tumors, *IQR* interquartile range, *AJCC* American Joint Committee on Cancer

### Statistical Analysis

Continuous variables were presented as median values (interquartile range [IQR]), and categorical variables were presented as frequency (%). Differences in baseline characteristics were assessed using the Kruskal–Wallis test for continuous variables and Chi-square or Fisher’s exact tests for categorical variables. Multivariable logistic regression analysis was performed to assess the adjusted probability of nodal metastasis (pN+) relative to MAI/SI in combination with the presence/absence of other risk factors. The prognostic impact of the extent of resection (i.e., RHC vs. appendectomy) among patients with MAI/SI and available follow-up data was assessed using the Kaplan–Meier method and the log-rank test. All tests were two-sided and statistical significance was assessed at α = 0.05. All analyses were performed using STATA version 18.0 (StataCorp LLC, College Station, TX, USA).

## Results

### Baseline Characteristics of All Patients Who Underwent Resection of Appendiceal Neuroendocrine Tumors

A total of 4819 patients underwent aNET resection and were included in the analytic cohort (Table 1). Median patient age was 51 years (IQR 34–63). Most patients were female (*n* = 2869, 59.5%), non-Hispanic White (*n* = 4164, 86.4%), and with a CCI of 0–1 (*n* = 4520, 93.8%). The majority of patients underwent simple appendectomy/segmental resection (*n* = 2910, 60.4%), while 31.1% (*n* = 1498) underwent RHC (total colectomy/other: *n* = 411, 8.5%). On final pathology, most patients had pT1 stage tumors (*n* = 2318, 48.1%) followed by pT3 stage tumors (*n* = 1662, 34.5%). Among patients with pT3 aNETs, 1309 (78.7%) had pT3a tumors (i.e., tumors ≤ 4 cm with MAI/SI), while 353 (21.2%) had pT3b tumors (i.e., tumors > 4 cm with or without MAI/SI) [ESM Table 2].

**Table 2:** Multivariable logistic regression analysis assessing factors associated with positive LN status (pN+) among patients with pT3a (i.e. +MAI/SI) aNETs

Variables	OR (95% CI)	*p*-Value
Age, years	0.98 (0.97–1.01)	0.062
Sex		
Female	Ref	
Male	1.01 (0.63–1.65)	0.938
Race		
Non-Hispanic White	Ref	
Non-Hispanic Black	1.22 (0.58–2.58)	0.587
Hispanic/other	0.25 (0.57–1.10)	0.066
Charlson-Deyo Score		
0	Ref	
1	0.70 (0.32–1.52)	0.373
≥ 2	0.50 (0.15–1.65)	0.259
Tumor size, cm		
< 1	Ref	
1–2	1.76 (0.76–4.08)	0.185
2–4	6.54 (2.84–15.1)	**<0.001**
Lymphovascular invasion		
No	Ref	
Yes	3.81 (2.25–6.46)	**<0.001**
Unknown	2.50 (0.99–6.30)	0.052
Tumor differentiation		
Well	Ref	
Moderate	0.47 (0.21–1.07)	0.074
Poor	2.14 (0.99–4.62)	0.053
Unknown	0.84 (0.42–1.67)	0.617
Type of surgery		
Appendectomy/segmental resection	Ref	
Right hemicolectomy	1.45 (0.79–2.64)	0.228
Total colectomy/other	2.50 (0.90–6.97)	0.080
Surgical margins		
*R*0	Ref	
*R*1/*R*2	6.76 (1.58–29.0)	**0.010**

Bold values denote statistical significance

*LN* lymph node, *MAI* mesoappendiceal invasion, *SI* subserosal invasion, *aNETs* appendiceal neuroendocrine tumors, *OR* odds ratio, *CI* confidence interval, *Ref* reference

### Incidence and Probability of pN+ Disease by pT Stage: The Role of Mesoappendiceal Invasion/Subserosal Invasion (MAI/SI)

**Fig. 1:**
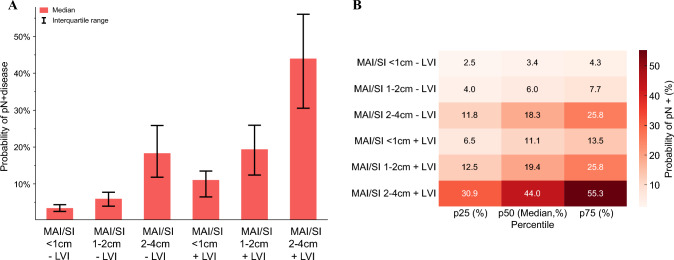
**a** Bar chart and **b** heatmap demonstrating the probability of pN+ disease among patients with pT3a aNETs according to the presence of risk factors (MAI/SI, tumor size, LVI). *aNETs* appendiceal neuroendocrine tumors, *MAI* mesoappendiceal invasion, *SI* subserosal invasion, *LVI* lymphovascular invasion

The overall incidence of pN+ disease in the entire cohort was 7.5% (*n* = 362) [pN0: *n* = 2084, 43.3%; pNx: 2373, 49.2%] and varied by AJCC pT stage (pT1: 0.9%; pT2: 9.2%; pT3: 8.5%; pT4: 29.8%; *p* < 0.001). Within pT3 stage (*n* = 1662), pT3a aNETs were less frequently associated with pN+ disease compared with pT3b tumors (6.8% vs. 14.7%; *p* = 0.02). To further delineate the association of MAI/SI with pN+ disease, a subgroup analysis was performed among patients with pT3a aNETs only (*n* = 1309) [i.e., tumors ≤ 4 cm and MAI/SI]. Among these patients, increasing tumor size (reference < 1 cm; 1–2 cm: odds ratio [OR] 1.76, 95% confidence interval [CI] 0.76–4.08, *p* = 0.185; 2–4 cm: OR 6.54, 95% CI 2.84–15.1, *p* < 0.001) and LVI (OR 3.81, 95% CI 2.25–6.46, *p* < 0.001) were the strongest predictors of pN+ disease (Table 2). Of note, in the absence of other established risk factors, the presence of MAI/SI alone was associated with a low probability of pN+ disease (3.4%) (Fig. 1).

### Extent of Resection in the Setting of MAI/SI

**Fig. 2:**
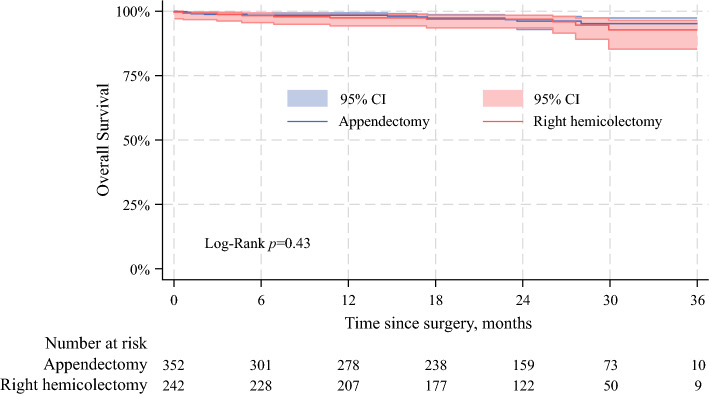
Kaplan–Meier curve demonstrating differences in OS between patients who underwent RHC versus simple appendectomy for pT3a aNETs. *OS* overall survival, *RHC* right hemicolectomy, *aNETs* appendiceal neuroendocrine tumors, *CI* confidence interval

Among individuals with pT3a aNETs (i.e., tumors ≤ 4 cm with MAI/SI), 720 (55.0%) received appendectomy/segmental resection, while 489 (37.4%) received RHC and 100 (7.6%) received total colectomy/other. Although RHC yielded a higher number of LNs than simple appendectomy (median 18 LNs [IQR 14–25] vs. 0 LNs [IQR 0–1]; *p* < 0.001), the median number of metastatic LNs among individuals with pT3 disease was similar (median 0 LNs [IQR 0–0] vs. 0 LNs [IQR 0–0]; *p* = 0.22). The 3-year OS among patients with pT3a aNETs with available follow-up data was comparable following RHC versus simple appendectomy (92.7% vs. 95.2%; *p* = 0.43) (Fig. 2).

## Discussion

An incidental aNET is a relatively common finding on postoperative pathology following appendectomy.^4,13^ While generally portending a favorable prognosis, the long-term outcomes are strongly influenced by LN status, which can generally only be determined after a staged RHC.^1,2,7^ Although multiple guidelines have typically recommended MAI as an indication for RHC among patients with aNETs measuring 1–2 cm,^7,9^ there is a paucity of data as to whether MAI alone, in the absence of other risk factors, warrants oncologic resection. Using a large cohort from the NCDB, the current study demonstrated that MAI/SI alone was associated with a low probability of nodal metastasis and that there was no difference in 3-year OS among patients who underwent RHC versus appendectomy alone for aNETs with MAI/SI, suggesting that RHC may not offer a clear survival benefit in this setting. These findings strongly suggest that the presence of MAI/SI alone, in the absence of other risk factors, should not be an indication for RHC.

Current literature is equivocal as to whether MAI is associated with a high risk for LN metastasis in aNETs.^11,14,15^ Most available studies comprise small, retrospective, heterogeneous cohorts, and therefore definitive conclusions regarding the prognostic impact of MAI cannot be safely drawn.^16^ Importantly, MAI can be observed in up to 20% of adults, however this information is often underreported.^11^ Indeed, MAI was only introduced as a staging factor in the 8th edition of the AJCC staging system (i.e., was not present in the 7th or earlier editions). As such, earlier studies did not account for this factor in a standardized manner.^17^ This change in staging criteria highlights the importance of contemporary studies that incorporate MAI into risk stratification models. In the present study, we leveraged the NCDB dataset restricted to cases after 2018, to capture information on MAI/SI. The current study demonstrated a nuanced relationship between MAI/SI and the probability of LN metastasis among patients with aNETs. While the presence of MAI/SI was associated with a low probability of pN+ disease in the absence of other risk factors (3.4%), the probability increased substantially in the presence of LVI or increasing tumor size. The data highlight the importance of taking into account all known risk factors when formulating the appropriate surgical plan for patients with aNETs. In addition, these data are important as they suggest that MAI/SI alone may not be sufficient to warrant RHC, particularly in the setting of small, low-risk aNETs. Indeed, the surgical approach and prognostic considerations for aNETs are distinct from those for appendiceal adenocarcinomas, which are typically managed similar to colon adenocarcinoma with RHC, regardless of MAI or tumor size.^18,19^

Current guidelines recommend completion RHC in the presence of aNETs sized >2 cm, incomplete resection (R1/R2), or aNETs 1–2 cm in size in the presence of additional ‘risk factors’ such as MAI, Ki-67 values >2%, and vessel invasion.^7,9^ Nevertheless, whether RHC offers a survival benefit over simple appendectomy for aNETs with MAI/SI remains controversial.^11,14,15,20^ Importantly, existing guidelines that recommend RHC in the setting of MAI have important limitations as they are based primarily on small, single-center, retrospective studies that often include heterogeneous patient populations, inconsistently report MAI depth, and lack long-term outcome data.^7,8^ This underscores the need for more robust prospective studies to clarify the true prognostic significance of MAI in aNETs. Even in the presence of aNETs >2 cm (an absolute criterion for RHC according to the existing guidelines),^6,7,9^ marked variations in surgical approach have been noted in the United States.^10^ In an NCDB analysis of 3198 cases with appendiceal carcinoids, Heller et al. reported that 32.4% of patients with tumors < 2 cm were treated with RHC, while 31.3% of individuals with tumors > 2 cm were treated with definitive appendectomy, highlighting the significant variations in practice and guideline non-adherence in the management of patients with aNETs.^10^ Moreover, a recent systematic review and meta-analysis of six studies involving 261 patients with aNETs reported that RHC could potentially be avoided for one in every five patients receiving oncologic resection, raising concerns for potential overtreatment.^21^ This is particularly relevant given that unlike for colorectal cancer, the pathologic findings from RHC (i.e., LN status) do not influence recommendations for adjuvant therapy. Furthermore, it is unlikely that removing microscopic disease as part of lymphadenectomy provides a survival benefit for early-stage NETs (and the number needed to treat would be very high even if it was beneficial). The current study showed that while the number of sampled LNs was higher following RHC versus simple appendectomy, the median number of metastatic LNs remained at zero across groups, suggesting limited additional oncologic yield with more extensive resection in this setting. In addition, the comparison of outcomes among patients with pT3a tumors (i.e., < 4 cm with MAI/SI) undergoing different surgical approaches (simple appendectomy vs. a more extensive RHC) revealed no significant survival benefit from RHC, supporting a more selective approach to extended resection. Therefore, the current study’s finding that RHC was not associated with a survival benefit is important evidence that this practice should be abandoned for patients with MAI alone.

The present study should be interpreted in light of certain limitations. Given the retrospective nature of this study, selection bias as to which patients received RHC versus simple appendectomy cannot be excluded. In addition, the NCDB does not specify which patients received appendectomy as the initial surgical procedure followed by completion RHC, limiting our ability to account for staged resection. Furthermore, MAI and SI were analyzed together, as the NCDB does not specifically allow for differentiation of the two pathologic features; however, since both are classified as pT3 in the 8th edition AJCC staging system, their prognostic significance is likely similar. Given that the definition of MAI was based on the 8th edition AJCC staging system, we were also unable to assess the prognostic impact of MAI above and below 3 mm. Data on Ki-67 and PNI were not available in the NCDB, which limited our ability to account for these variables in the current analysis. Finally, the current study was limited to cases after 2018 (to leverage data from the 8th edition AJCC staging system), which limited the duration of available follow-up in our cohort.

## Conclusion

In this large NCDB cohort of patients with aNETs, MAI/SI alone in the absence of other established risk factors was associated with a low likelihood of nodal metastasis. RHC did not appear to confer a survival benefit over simple appendectomy among patients with +MAI/SI aNETs. These data provide critical insights into the management of aNETs, suggesting that MAI/SI alone should not be an absolute indication for RHC. Given the risk of overtreatment, additional research including multi-institutional prospective trials are warranted to further refine risk stratification and optimize treatment recommendations for patients with aNETs.

## Supplementary Information

Below is the link to the electronic supplementary material.

Supplementary file1 (DOCX 136 KB)
