# Supplementary material for: Appendiceal Neuroendocrine Tumors: Prognostic Role of Mesoappendiceal Invasion and Implications for Recommending Right Hemicolectomy versus Simple Appendectomy
Source: Ann Surg Oncol. 2025 Aug 5;32(10):7627–33. doi: 10.1245/s10434-025-17982-7 (PMC12454516; doi:10.1245/s10434-025-17982-7)
Supplement: Supplementary file 1 — Supplementary file1 (DOCX 136 KB) [file 10434_2025_17982_MOESM1_ESM.docx]

**Supplemental Table 1** Summary of pT stage classification of aNET according to AJCC/UICC staging schema and classification used in this study to delineate the role of MAI/SI.

| **Pathologic T stage** | **AJCC/UICC**  **7^th^ edition** | **AJCC/UICC 8^th^ edition** | **Classification used in this study** |
| --- | --- | --- | --- |
| **pT1** | < 2 cm (T1a < 2cm; T1b 1-2cm | < 2cm | Same as AJCC 8^th^ pT1 |
| **pT2** | > 2-4 cm OR cecal invasion | > 2-4cm | Same AJCC 8^th^ pT2 |
| **pT3** | >4cm or ileal invasion | > 4cm OR mesoappendix / subserosa invasion | **T3a: < 4cm AND mesoappendix/subserosal invasion***  **T3b: > 4cm AND/OR mesoappendix/subserosal invasion** |
| **pT4** | Perforates serosa/peritoneum or invades other neighboring organs | | |

aNET: appendiceal neuroendocrine tumor; AJCC/UICC: American Joint Committee on Cancer / International Union Against Cancer; MAI/SI: mesoappendiceal invasion / subserosal invasion

* Information on MAI/SI can only be identified in the NCDB using the AJCC 8^th^ edition staging system. According to that, pT3 stage includes tumors > 4 cm OR tumors with MAI/SI. In turn, pT3 tumors with size < 4 cm – by definition - have MAI/SI (to be staged as pT3). In the context of this study, this group of tumors with definitive presence of MAI/SI on pathology was categorized as pT3a. The remaining pT3 aNETs with size > 4 cm may or may not have MAI/SI as the size by itself would fulfill the criteria to be staged as pT3 tumors – this group was categorized as T3b in our study. This categorization allowed us to isolate the MAI/SI component and delineate the role of MAI/SI relative to the probability of pN+ disease.

**Supplemental Table 2** Clinicopathologic characteristics of patients with pT3a versus pT3b aNETs

| **Variables** | **All pT3**  **(n=1,662)** | **pT3a**  **(n=1,309, 78.8%)** | **pT3b**  **(n=353, 21.2%)** | **P-value** |
| --- | --- | --- | --- | --- |
| Age, years (IQR) | 53 (37-63) | 51 (34-63) | 57 (45-65) | <0.001 |
| Sex |  |  |  | 0.338 |
| Female | 974 (58.6%) | 775 (59.2%) | 199 (56.4%) |  |
| Male | 688 (41.4%) | 534 (40.8%) | 154 (43.6%) |  |
| Race |  |  |  |  |
| Non-Hispanic White | 1,429 (86.0%) | 1,127 (86.1%) | 302 (85.6%) | 0.394 |
| Non-Hispanic Black | 146 (8.8%) | 110 (8.4%) | 36 (10.2%) |  |
| Hispanic / Other | 87 (5.2%) | 72 (5.5%) | 15 (4.2%) |  |
| Charlson-Deyo Score |  |  |  |  |
| 0 | 1,374 (82.7%) | 1,092 (83.4%) | 282 (79.9%) | 0.240 |
| 1 | 190 (11.4%) | 141 (10.8%) | 49 (13.9%) |  |
| >= 2 | 98 (5.9%) | 76 (5.8%) | 22 (6.2%) |  |
| Facility Type |  |  |  |  |
| Community Cancer Program | 109 (9.2%) | 81 (9.2%) | 28 (9.2%) | 0.726 |
| Comprehensive Community Cancer Program | 456 (38.4%) | 340 (38.4%) | 116 (38.3%) |  |
| Academic / Research Program | 348 (29.3%) | 253 (28.6%) | 95 (31.4%) |  |
| Integrated Network Cancer Program | 275 (23.1%) | 211 (23.8%) | 64 (21.1%) |  |
| Not available | 456 (38.4%) | 340 (38.4%) | 116 (38.3%) |  |
| Tumor size |  |  |  |  |
| <1 cm | 466 (28.0%) | 466 (35.6%) | 0 (0.0%) | <0.001 |
| 1-2 cm | 607 (36.5%) | 607 (46.4%) | 0 (0.0%) |  |
| 2-4 cm | 236 (14.2%) | 236 (18.0%) | 0 (0.0%) |  |
| >4cm | 184 (11.1%) | 0 (0.0%) | 184 (52.1%) |  |
| Unknown | 169 (10.2%) | 0 (0.0%) | 169 (47.9%) |  |
| pN status |  |  |  |  |
| pN0 | 819 (49.3%) | 593 (45.3%) | 226 (64.0%) | <0.001 |
| pN+ | 134 (8.1%) | 86 (6.6%) | 48 (13.6%) |  |
| Unknown/ not examined | 709 (42.7%) | 630 (48.1%) | 79 (22.4%) |  |
| Number of sampled nodes (IQR) | 10 (0-19) | 2 (0-19) | 15 (3-22) | <0.001 |
| Number of positive nodes (IQR) | 0 (0-0) | 0 (0-0) | 0 (0-0) | 0.047 |
| Lymphovascular invasion | 276 (16.6%) | 204 (15.6%) | 72 (20.4%) | <0.001 |
| Tumor differentiation |  |  |  |  |
| Well | 1,029 (61.9%) | 888 (67.8%) | 141 (39.9%) | <0.001 |
| Moderate | 201 (12.1%) | 143 (10.9%) | 58 (16.4%) |  |
| Poor | 116 (7.0%) | 73 (5.6%) | 43 (12.2%) |  |
| Unknown | 316 (19.0%) | 205 (15.7%) | 111 (31.4%) |  |
| Type of surgery |  |  |  |  |
| Appendectomy / Segmental resection | 859 (51.7%) | 720 (55.0%) | 139 (39.4%) | <0.001 |
| Right hemicolectomy | 686 (41.3%) | 489 (37.4%) | 197 (55.8%) |  |
| Total colectomy / Other | 117 (7.0%) | 100 (7.6%) | 17 (4.8%) |  |
| Surgical margins |  |  |  |  |
| R0 | 1,576 (94.8%) | 1,253 (95.7%) | 323 (91.5%) | 0.006 |
| R1/R2 | 65 (3.9%) | 42 (3.2%) | 23 (6.5%) |  |
| Unknown | 21 (1.3%) | 14 (1.1%) | 7 (2.0%) |  |
